# Case Report: Minimal Neurological Deficit of Two Adult Patients With Weston–Hurst Syndrome Due to Early Craniectomy: Case Series and Review of Literature on Craniectomy

**DOI:** 10.3389/fneur.2021.673611

**Published:** 2021-08-31

**Authors:** Anna Mira Loesch-Biffar, Andreas Junker, Jennifer Linn, Niklas Thon, Suzette Heck, Caroline Ottomeyer, Andreas Straube, Hans Walter Pfister

**Affiliations:** ^1^Department of Neurology, University Hospital, Ludwig-Maximilians-University, Munich, Germany; ^2^Institute of Neuropathology, University Hospital, Ludwig-Maximilians-University, Munich, Germany; ^3^Department of Radiotherapy and Radiation Oncology, Faculty of Medicine and University Hospital Carl Gustav Carus, Technische Universität Dresden, Dresden, Germany; ^4^Department of Neurosurgery, University Hospital, Ludwig-Maximilians-University, Munich, Germany

**Keywords:** Weston-Hurst syndrome, decompressive craniectomy, outcome, autoimmun disease, acute hemorrhagic leukoencephalitis

## Abstract

**Objectives:** We describe two new cases of acute hemorrhagic leucoencephalitis (AHLE), who survived with minimal sequelae due to early measures against increased intracranial pressure, particularly craniotomy. The recently published literature review on treatment and outcome of AHLE was further examined for the effect of craniotomy.

**Methods:** We present two cases from our institution. The outcome of 44 cases from the literature was defined either as good (no deficit, minimal deficit/no daily help) or poor outcome (severe deficit/disabled, death). Patients with purely infratentorial lesions (*n* = 9) were excluded. Fisher's exact test was applied.

**Results:** Two cases are presented: A 43-year-old woman with rapidly progressive aphasia and right hemiparesis due to a huge left frontal white matter lesion with rim contrast enhancement. Pathology was consistent with AHLE. The second case was a 56-year-old woman with rapidly progressive aphasia and right hemiparesis. Cranial MRI showed a huge left temporo-occipital white matter lesion with typical morphology for AHLE. Both patients received craniotomy within the first 24 h and consequent immunosuppressive-immunomodulatory treatment and survived with minimal deficits. Out of 35 supratentorial reported AHLE cases, seven patients received decompressive craniotomy. Comparing all supratentorial cases, patients who received craniotomy were more likely to have a good outcome (71 vs. 29%).

**Conclusion:** Due to early control of the intracranial pressure, particularly due to early craniotomy; diagnosis per biopsy; and immediate start of immunosuppressive-immunomodulatory therapies (cortisone pulse, plasma exchanges), both patients survived with minimal sequelae. Craniotomy plays an important role and should be considered early on in patients with probable AHLE.

## Introduction

Acute hemorrhagic leucoencephalitis (Weston–Hurst, AHLE) is a rare, rapidly progressive, demyelinating disease, commonly considered to be a severe variant of an acute disseminated encephalomyelitis (ADEM) (1, 2). The disease is often preceded by a respiratory tract infection (3–5), and in some cases, AHLE also occurred shortly after seasonal influenza vaccinations (6, 7). Most patients die within a couple of days due to fulminant brain edema. It more commonly affects the supratentorial regions, but there are also cases documented with infratentorial lesions (6, 8–10). Mortality is still high, but there are cases in the literature with a good outcome (11–14). There are no standard operating procedures concerning the treatment (12). A detailed systematic review from Grzonka (15) shows that most of the patients were treated immunosuppressive-immunomodulatory, mostly with glucocorticoids. There was no relationship between the delay between the start of the treatment and the outcome. With our two successfully treated fulminant AHLE cases, our aim was, in combination with the reviewed cases from the literature, to find out what may have attributed to the good outcome.

## Methods

We present two cases from our institution with AHLE seen in the last 7 years. The systematically reviewed AHLE case collective, including the presented case of Grzonka et al. (15), was examined for their outcomes, depending on whether they were treated with craniotomy. Purely infratentorial localized cases were excluded.

Outcome was defined either as no deficit, minimal deficit (no influence on everyday life), severe deficit (disabled), or death. Fisher's exact test was applied.

## Results

### Case Presentation

#### Case 1

**Figure 1:**
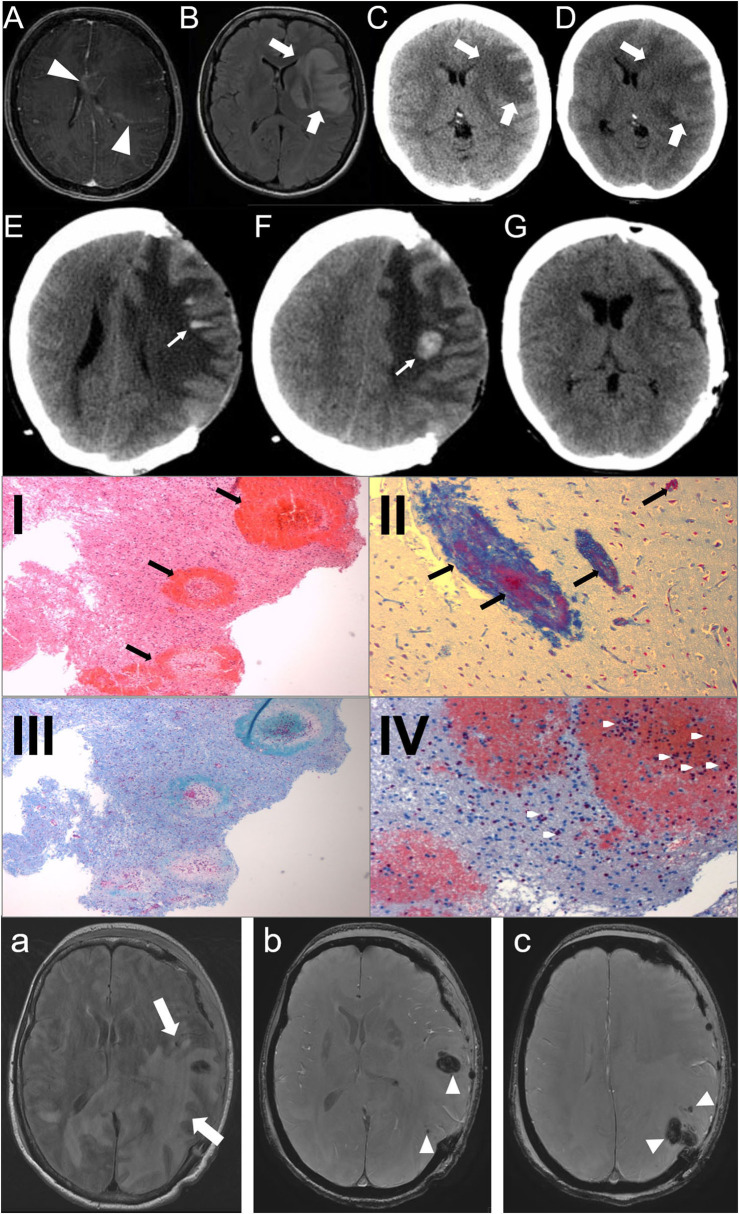
**(A–G)**: Case 1: Day 1: axial MRI, T1-weighted with contrast **(A)**, FLAIR-sequence **(B)**, CT-scan **(C)** show a left frontal white matter lesion (big arrows) with rim contrast enhancement (arrowheads) and midline shift; Day 2 **(D)**: CT-scan: progressive edema; Day 15 **(E,F)**: CT-scan: bleedings (small arrows); Day 80 **(G)**: reimplantation of bone plate. **(I–IV)**: Histopathology of the brain biopsy (Case 1). Multiple perivascular bleedings [**(I)**, HE-stain], fibrin accumulation [**(II)**, Ladewig-stain], demyelinization [**(III)**, LFB-PAS] and granulocystic infiltration [**(IV)**, Chloracetatesterase]. **(a–c)**: Case 2: Day 3: axial MRI, FLAIR-sequence **(a)** shows a subcortical left temporooccipital white matter lesion (big arrows) with midline shift and multiple hemorrhages (arrowheads) in SWAN-sequence **(b,c)**.

A 43-year-old woman with a preceding unspecific respiratory infection was admitted to the hospital due to rapidly progressive aphasia and right hemiparesis. Brain MRI revealed a left frontal white matter lesion with rim contrast enhancement (Figures 1A,B). In the cerebrospinal fluid, 112 cells/μl (granulocytic) and oligoclonal IgG-bands were found. Neuroinfectious pathogens were not detected. Under the suspected diagnosis of an ADEM, glucocorticoid pulse therapy was initiated (1 g per day). As the patient still rapidly worsened and imaging revealed progressive edema with a midline shift (Figures 1C,D), hemicraniectomy was performed (<24 h after admission). Histopathology of cortical biopsy was consistent with AHLE (Weston–Hurst, Figures 1I–IV). The patient sequentially received another cortisone pulse (methylprednisolone 1 g for 5 days), immunoglobulins (120 g for 5 days), and seven cycles of therapeutic plasma exchange until day 36 after symptom onset. Under this treatment, the maximum of the swelling was reached on day 15 (Figures 1E,F), from that time on, the patient improved in both the imaging finding and clinically. Four months after symptom onset, the bone flap could be reimplanted (Figure 1G). The patient survived with a minimal weakness of the right hand and slight neurocognitive deficits.

#### Case 2

The second case was a 56-year-old woman who also presented with aphasia and right hemiparesis. She also had a history of a recent unspecific respiratory infection over the last days. CT scan of the brain revealed a huge left temporo-occipital lesion with midline shift. As the patient rapidly worsened and the lesion was progressive in the CT scan, the patient immediately (within 24 h after admission) underwent decompressive craniotomy. Cranial MRI also showed a huge left temporo-occipital white matter lesion with multiple small hemorrhages. The lesion was located predominantly subcortical without affecting the temporal pole, typical for AHLE (Figures 1a-c). Due to the rapid course of the disease and the morphology of the lesion, AHLE was immediately suspected. Glucocorticoid pulse therapy was initiated (methylprednisolone 1 g for 5 days) followed by tapering with prednisolone, immunoglobulins (20 g/day for 5 days) and six cycles of immunoabsorption. The bone flap was reimplanted 4 months after symptom onset. The patient also survived with minimal sequelae.

In the literature from January 1, 2000, until the publication of Grzonka et al. in 2020, 44 adult patients are reported. One case with a spinal localization of the lesion was excluded. Out of 43 cases, 30 are males (70%) and 13 females (30%); the mean age was 38 years. Eight patients had only infratentorial lesions (seven males, 88%; one female, 12%, mean age 44 years). Out of 35 supratentorial reported AHLE cases [23 males, 66%, 12 females, 34%, mean age 38 years; Table 1; (1, 2, 5, 7–46)], seven patients received decompressive craniotomy [Table 2; (2, 5, 11–13, 18, 42)]. In four of the operated patients, hemorrhages were detected in neuroimaging prior to surgery (2, 11, 13, 42). In the other patients, the diagnosis of AHLE was confirmed by biopsy (12, 18) or postmortem (5). Patients who received craniotomy, like our two cases, were more likely to have a good outcome (71%, *n* = 5; Table 3, Fisher's exact test: *p* = 0.0754). Three of the seven patients who received craniotomy had no deficit, two had minimal sequelae, and only two patients died (29%). The initial biopsy of one of those two patients (5) showed a neutrophil predominance and did not lead to the direct diagnosis of AHLE. Thus, a differential diagnosis of pneumonia with hematogenous spread to the brain was suspected, and the craniectomy was combined with antibiotics instead of a combination with immunosuppressive therapy. The other patient who died despite craniectomy had a 2-week history of illness and refused to go to the hospital at first (42). Also, the MRI could not be obtained on the first day after admission, which led to a delay in immunosuppressive treatment. In the non-operated group, 71% (*n* = 20) of the patients had an unfavorable outcome (7 patients severely disabled, 13 died), and 29% survived with minimal (*n* = 6) to no deficit (*n* = 2). This difference, however, just did not reach statistical significance (Fisher's exact test: p = 0.0754).

**Table 1:** Clinical, neuroradiologic, and neuropathologic characteristics of adult patients with AHLE.

**(Ref. No.), Year of publication**	**Age**	**Sex**	**Localization of lesions associated with AHLE**	**Unilateral or bilateral**	**Immunosuppressive-immunomodulatory treatment**	**Diagnosis per biopsy**	**Decompressive craniotomy**	**Outcome**
(14), 2000	34	Male	Supratentorial	Bilateral	Dexamethasone 24 mg/d	No	No	Minimal deficit
(16), 2001	41	Female	Supra- and infratentorial	Bilateral	Post mortem diagnosis	Post mortem	No	Death
(17), 2001	44	Female	Supratentorial	Bilateral	Dexamethasone 15 mg/d, Methylprednisolone 1 g/d	No	No	Minimal deficit
(18), 2002	28	Male	Supratentorial	Unilateral	Glucocorticoids	Yes	Yes	Minimal deficit
(19), 2003	19	Male	Supratentorial	Unilateral	Dexamethasone	Yes	No	Death
(20), 2004	57	Female	Supratentorial	Bilateral	Methylprednisolone 1 g/d	No	No	Severe deficit
(21), 2004	28	Male	Supratentorial	Bilateral	Dexamethasone 16 mg/d	Yes	No	Death
(22), 2005	43	Male	Supratentorial	Bilateral	Dexamethasone	Yes	No	Minimal deficit
(23), 2005	42	Female	Supra- and infratentorial	Bilateral	Prednisolone	Yes	No	Minimal deficit
(24), 2006	22	Female	Supratentorial	Bilateral	Methylprednisolone 1 g/d	No	No	Minimal deficit
(12), 2007	31	Male	Supratentorial	Unilateral	Dexamethasone, cortisone pulse therapy, plasmapheresis	Yes	Yes	No deficit
(25), 2009	30	Male	Supra- and infratentorial	Bilateral	Methylprednisolone	No	No	Death
(26), 2009	62	Male	Supra- and infratentorial	Unilateral	Dexamethasone 32 mg/d, plasmapheresis	No	No	Severe deficit
(11), 2010	20	Male	Supratentorial	Unilateral	Not reported	Yes	Yes	No deficit
(27), 2010	76	Male	Infratentorial	–	Methylprednisolone 500 mg/d, tapering	No	No	Minimal deficit
(28), 2010	40	Male	Supratentorial	Bilateral	Cortisone, plasmapheresis	No	No	Severe deficit
(9), 2010	21	Male	Infratentorial	–	Methylprednisolone 1 g/d, plasmapheresis	No	No	Death
(29), 2011	25	Male	Supratentorial	Unilateral	Methylprednisolone	Yes	No	Severe deficit
(30), 2011	56	Female	Supratentorial	Bilateral	Methylprednisolone 500 mg/d	No	No	Minimal deficit
(31), 2011	70	Male	Supra- and infratentorial	Bilateral	Methylprednisolone 1 g/d, immunoglobulines	No	No	Death
(32), 2011	37	Male	Infratentorial	–	Glucocorticoids	Yes	No	Minimal deficit
(33), 2011	23	Male	Infratentorial	–	Methylprednisolone 1 g/d, followed by prednisolone 40 mg/d	No	No	Death
(34), 2012	51	Male	Infratentorial	–	Dexamethasone	No	No	Death
(35), 2013	27	Male	Supra- and infratentorial	Bilateral	Not reported	Postmortem	No	Death
(36), 2013	22	Male	Supratentorial	Bilateral	Prednisolone	Postmortem	No	Death
(8), 2014	75	Male	Infratentorial	–	Not reported	Postmortem	No	Death
(37), 2014	39	Male	Supra- and infratentorial	Bilateral	Not reported	Postmortem	No	Death
(13), 2014	24	Female	Supratentorial	Unilateral	Dexamethasone, plasmapheresis	Yes	Yes	No deficit
(38), 2015	48	Male	Supra- and infratentorial	Bilateral	Not reported (found death)	Postmortem	No	Death
(1), 2015	21	Female	Supratentorial	Bilateral	Prednisolone	Yes	No	Severe deficit
(10), 2016	34	Female	Infratentorial	–	Methylprednisolone, immunoglobulines	No	No	Death
(5), 2016	27	Male	Supratentorial	Unilateral	Dexamethasone, 12 mg/d	Yes (but unspecific cerebritis)	Yes	Death
(39), 2016	44	Male	Supratentorial	Bilateral	Not reported	No	No	No deficit
(40), 2016	25	Female	Supra- and infratentorial	Bilateral	Methylprednisolone 1 g/d, plasmapheresis	Not reported	No	Severe deficit
(41), 2017	33	Female	Supratentorial	Unilateral	Dexamethasone 16 mg/d for 14 days, tapering for 2 months	Yes	No	No deficit
(42), 2017	19	Male	Supratentorial	Unilateral	Methylprednisolone for 1 day, immunoglobulins, rituximab, cyclophosphamide, plasmapheresis	No	Yes	Death
(43), 2017	36	Female	Supratentorial	Unilateral	Methylprednisolone 1 g several days, plasmapheresis	No	No	Death
(44), 2017	36	Male	Infratentorial	–	Not reported	No	No	Death
(7), 2018	70	Male	Supra- and infratentorial	Bilateral	Methylprednisolone 1 g, plasmapheresis	No	No	Death
(2), 2018	25	Female	Supra- and infratentorial	Bilateral	Glucocorticoids 1 g/d for 5 days, plasmapheresis	No	Yes	Minimal deficit
(45), 2019	63	Male	Supratentorial	Bilateral	Methylprednisolone 1 g/d for 5 days, Dexamethasone 0.15 mg/kg body weight/d	No	No	Severe deficit
(46), 2019	42	Male	Supra- and infratentorial	Unilateral	Not reported	Postmortem	No	Death
(15), 2020	59	Male	Supra- and infratentorial	Bilateral	Methylprednisolone 2 g/d for 3 days, tapering, immunoglobulins, cyclophosphamide	Yes	No	Death

**Table 2:** Clinical, neuroradiologic, and neuropathologic characteristics of adult patients who received craniectomy with AHLE.

**(Ref. No.), Year of publication**	**Age**	**Sex**	**Localization of lesions associated with AHLE**	**Unilateral or Bilateral**	**Hemorrhages in neuroimaging associated with AHLE prior to surgery**	**Immunosuppressive-immunomodulatory treatment**	**Diagnosis per biopsy**	**Approximate time of craniectomy after admission**	**Outcome**	**Particularities of the case presumably leading to poor outcome**
(18), 2002	28	Male	Supratentorial	Unilateral	Not reported	Glucocorticoids	Yes	Day 1	Minimal deficit	–
(12), 2007	31	Male	Supratentorial	Unilateral	Not reported	Dexamethasone, cortisone pulse therapy, plasmapheresis	Yes	Day 5	No deficit	–
(11), 2010	20	Male	Supratentorial	Unilateral	Progressive lesion with hemorrhagic change	Not reported	Yes	Day 2	No deficit	–
(13), 2014	24	Female	Supratentorial	Unilateral	Mass lesion with abnormal signal and multiple small hemorrhages	Dexamethasone, plasmapheresis	Yes	Day 1	No deficit	–
(5), 2016	27	Male	Supratentorial	Unilateral	Not reported	Dexamethasone, 12 mg/d	Yes (but showed unspecific cerebritis)	Day 2	Death	AHLE only diagnosed postmortem → early surgery but only a low dose of glucocorticoids
(42), 2017	19	Male	Supratentorial	Unilateral	Asymmetric lesion with hemorrhage evident on gradient recalled echo sequences along with edema	Methylprednisolone for 1 day, immunoglobulins, rituximab, cyclophosphamide, plasmapheresis	No	Day 2	Death	2 weeks of malaise, refusing to go to hospital, initial MRI could not be obtained → time delay
(2), 2018	25	Female	Supra- and infratentorial	Bilateral	Extensive edema with multiple punctiform bleeding	Glucocorticoids 1 g/d for 5 days, plasmapheresis	No	Day 1	Minimal deficit	–

**Table 3:** Statistical analysis of the outcome of adult AHLE patients regarding craniectomy, Fisher's exact test, *p*-value = 0.0754.

	**Good outcome**	**Poor outcome**
Craniectomy	*N* = 5 (*n* = 3 with no deficit; *n* = 2 with minimal sequelae)	*N* = 2 (died)
No Craniectomy	*N* = 8 (*n* = 2 with no deficit; *n* = 6 with minimal sequelae)	*N* = 20 (*n* = 7 severely disabled; *n* = 13 died)

## Discussion

AHLE is still a rare and fulminant disease with a mostly life-threatening outcome and a high mortality. The disease mostly affects young males but is also reported in patients of all ages (16). Even with early aggressive immunosuppressive treatment, AHLE can be a devastating condition in terms of mortality and severe neurological sequelae. One of the most detailed reviews on AHLE by Grzonka et al. (15) shows that, looking only at immunosuppressive and immunomodulatory treatments, there was no clear relationship between different treatments and outcome.

Thus, based on our literature review, we consider that, early interventions against the increased intracranial pressure due to the rapid increasing brain edema, especially craniotomy, can change the fulminant course of the disease. We suggest that better prognosis can be expected when craniotomy is performed early, together with a consequent immunosuppressive-immunomodulatory treatment at the same time than medical treatment alone. Despite the worry that sudden decompression could aggravate intracerebral bleedings of the hemorrhagic encephalitis, the mass effect of the disease seems to be the life-limiting factor. This is in line with a review on decompressive craniotomy in viral encephalitis patients with brain herniation (47) and also in patients with spontaneous intracerebral hemorrhage (48). They also suggest that, in those cases, a better prognosis without increasing the hemorrhage can be expected when craniotomy is performed in addition to medical treatment alone.

We believe that the present data support the decision for an early surgical decompression in patients with severe and rapid AHLE and should be considered early on. Control of the intracranial pressure seems to be an important part of the therapy concept in addition to early immunosuppression. Still, this is the conclusion based on a review of literature with a relative low number of cases and, therefore, only a low level of evidence. Otherwise, we don't expect that, in such a rare and severe disease, a prospective controlled study can be done.

## Data Availability Statement

The raw data supporting the conclusions of this article will be made available by the authors, without undue reservation.

## Ethics Statement

Written informed consent was obtained from the individual(s) for the publication of any potentially identifiable images or data included in this article.

## Author Contributions

AL-B contributed by executing the literature search, collected the data, and drafted and revised the manuscript. AJ, JL, NT, SH, CO, and AS helped with the data collection and manuscript revision. AJ also contributed with the histopathological images. JL provided the radiological images. AS also helped with the literature search. HP interpreted the data, helped with the literature search, and revised the manuscript for intellectual content. All authors contributed to the article and approved the submitted version.

## Conflict of Interest

The authors declare that the research was conducted in the absence of any commercial or financial relationships that could be construed as a potential conflict of interest.

## Publisher's Note

All claims expressed in this article are solely those of the authors and do not necessarily represent those of their affiliated organizations, or those of the publisher, the editors and the reviewers. Any product that may be evaluated in this article, or claim that may be made by its manufacturer, is not guaranteed or endorsed by the publisher.
